# Managing Transition in Patients Treated with Growth Hormone

**DOI:** 10.3389/fendo.2017.00346

**Published:** 2017-12-11

**Authors:** Berthold P. Hauffa, Philippe Touraine, Tanya Urquhart-Kelly, Ekaterina Koledova

**Affiliations:** ^1^Department of Pediatric Endocrinology, University of Duisburg-Essen, Essen, Germany; ^2^Department of Endocrinology and Reproductive Medicine, Centre des Maladies Endocriniennes Rares de la Croissance et du Développement, Paris, France; ^3^Department of Nursing and Midwifery, Sheffield Hallam University, Sheffield, United Kingdom; ^4^Global Medical Affairs, Merck KGaA, Darmstadt, Germany

**Keywords:** growth hormone deficiency, pediatric, adolescent, transition, personalized medicine, endocrinology

## Abstract

Growth hormone (GH) promotes growth in children, but is also essential for bone strength, body composition, metabolic factors, such as lipid profile, and maintenance of quality of life. The Merck KGaA (Germany) funded “360° GH in Europe” meeting, held in Lisbon, Portugal, in June 2016, comprised three sessions entitled “*Short Stature Diagnosis and Referral*,” “*Optimizing Patient Management and Adherence*,” and “*Managing Transition*.” The scientific program covered all stages of pediatric GH treatment, and reported here are the outcomes of the third session of the meeting, which considered transition from pediatric GH treatment to teenage and young adult GH therapy. A large number of patients with chronic diseases, including GH deficiency, drop out of therapy during the transition period. Multiple factors are associated with this, such as lack of understanding of the disease process, insufficient knowledge of treatment options, the patient becoming more independent, and requirement for interaction with a new set of health-care workers. Education regarding disease management and treatment options should be provided from an early age and right through the transition period. However, endocrine specialists will view the transition period differently, depending on whether they are pediatric endocrinologists who mainly deal with congenital diseases, in which auxology is important, or adult endocrinologists who are more concerned with body composition and metabolic factors. View points of both a pediatric and an adult endocrine specialist are presented, together with a case study outlining practical aspects of transition. It was noted in the meeting discussion that having one person to guide a patient through transition from an early age is important, but may be constrained by various factors such as finances, and options will differ by country.

## Introduction

Growth hormone (GH) not only promotes growth in children but also develops bone strength, maintains lean body mass and muscle strength while reducing fat mass, normalizes metabolic factors such as lipid profile, and helps to maintain quality of life (1–3). Somatic development continues beyond adolescence, and the effects of GH on body composition and metabolism continue into adult life (4). However, a proportion of patients with GH deficiency identified during childhood have sufficient GH secretion when stimulation tests are repeated during adolescence or early adulthood (5). Therefore, GH-deficient children who receive pediatric GH treatment require monitoring of GH status during adolescence, after final height is considered to have been achieved (6, 7). Algorithms have been proposed for such re-assessment of GH status at the transition period (7), but remain equivocal. For patients who are still identified as GH deficient, continuing GH treatment into adulthood is required, although at a lower dose than during childhood, and GH treatment for adults with GH deficiency is an approved indication (3). While continued GH treatment is necessary during the time from pediatric status to teenage and young adult age, this transition can be a difficult period and requires consideration of many factors. Both pediatric and adult endocrine specialists need to be involved, as well as the patient, their family and other members of the health-care team. Specific transition clinics that guide the patients through this period appear to help to improve various aspects of the process (8, 9). The present report from the third session of the 360° GH in Europe meeting considers aspects of transition from pediatric GH treatment to young adult GH treatment from different points of view of endocrine specialists. Reports from the other sessions on diagnosis and referral and on optimizing patient management are published separately (10).^1^

## Transition from the Perspective of a Pediatric Endocrinologist

Transition can be defined from physiological changes during the period from the end of puberty to the attainment of an adult phenotype. The start of transition may be considered to be when patients reach Tanner stage 5, reported to occur at a mean age of 14.7 ± 2.2 years in boys or 14.0 ± 2.4 years in girls (11). It can also be considered to be when patients have reached their final adult height, at the mean age of 16.8 ± 2.2 years in boys and 15.2 ± 2.0 years in girls (11). The end of transition can be determined from sleep chronotype when there is a switch in time of going to bed, which occurs at a mean of 20.9 years for males or 19.5 years for females (12), or from reaching peak bone mass, reported to occur at a mean of 23.1 years in males and 19.9 years in females (13). This gives the length of transition as approximately 6–9 years for boys and 5–6 years for girls, although it may be longer or shorter depending on multiple factors.

Alternatively, transition can be seen as the point in time when the patient can no longer be seen by the pediatrician and is asked to make the next appointment with an adult endocrinologist. However, focusing on a single time point is not good for a patient receiving GH because the transition from pediatric treatment to adult treatment needs to be structured. In a study of UK patients with congenital adrenal hyperplasia (14), which is diagnosed during the neonatal period, the number of patients being treated as adults by specialist endocrine centers was only about 10% of the number expected based on prevalence. Such studies indicate a high risk for adolescents to drop out of specialist endocrine care during transition to adulthood.

Many factors influence the drop out of patients during transition, including the differences between pediatric and adult treatment modalities. Pediatric endocrinologists predominantly deal with congenital diseases and with diseases in which auxology is important, whereas adult endocrinologists are mostly treating acquired diseases and auxology is rarely important. Data analysis for children requires age-matched reference information, but reference data are constant over a wide age range in adults. Assay methodologies differ, with special requirements in children but more standardized in adults, and cutoffs for endocrine tests are very different. Young children are less able to understand diseases and therapies, requiring parents or guardians to help them, whereas adults are much more involved in decision-making. Thus, there are differences in interactions with the patients, whereby pediatric endocrinologists are more likely to encourage patients through transition and push for interaction with adult endocrinologists, who may take a more wait-and-see approach.

Growth hormone replacement in children with GH deficiency has been shown to be effective in normalizing growth and achieving adult height within their genetic target range (15). In contrast, GH treatment of deficiency in adults is associated with improvements in body composition, muscle strength, lipid profile, cardiovascular risk, and quality of life (6, 16). For patients who are GH deficient during adulthood, continuing GH treatment is important for body homeostasis. However, a majority of patients who require GH treatment for idiopathic or non-organic GH deficiency during childhood do not remain GH deficient when re-tested after treatment completion (5, 6, 16). Therefore, appropriate re-assessment is mandatory to identify patients requiring lifelong GH therapy.

An algorithm for management of patients during transition after treatment with GH during childhood was proposed in a consensus statement from the European Society of Paediatric Endocrinology (ESPE) (7). When height velocity has decreased to <1.5/2.0 cm/year, GH should be discontinued for 1–3 months. Patients should then be grouped as either high likelihood of continuing to be GH deficient, with severe deficiency due to genetic or organic causes and particularly with multiple pituitary hormone deficiencies, or low likelihood, including patients with idiopathic GH deficiency that is either isolated or with only one additional pituitary hormone deficiency. Patients with high likelihood who then have an IGF-I SDS ≤−2 should restart GH; if IGF-I is >−2 SDS then a stimulation test should be performed and GH treatment should only be re-started if the stimulated peak is below the reference cutoff. Patients with low likelihood should have both IGF-I assessment and a GH stimulation test, with GH re-started if both are low indicating continued GH deficiency; if both are normal then GH deficiency is excluded at that time and if the results are discordant then the patient should continue follow-up.

While this algorithm provided some consensus, evidence is lacking in many instances. The cutoff for adult patients for the insulin tolerance stimulation test was set as <3 μg/l by ESPE and also the GH Research Society (GRS), but at <5 μg/l by the American Association of Clinical Endocrinology and the Endocrine Society. Similarly, different cutoff values have been proposed for the GH releasing hormone–arginine stimulation test, and the GRS also proposed different cutoff values depending on adult body mass index. These proposed values are for measurements in adults, but have not been established for patients in the transition period. This has led to widely varying clinical practice in establishing the need for continued treatment and the way that continuing GH deficiency is managed (17, 18). Therefore, new guidelines are required, which should address the unresolved issues.

## Transition from the Perspective of an Adult Endocrinologist

Transition should not be seen as a simple move from one site to another, but is a multi-step process during which the medical, psychological, social, and educational needs must all be considered (19, 20). Therefore, it is necessary to work with the patient as a young adult, with implementation steps through transition organized with the active participation of the patient. As a result, there are many obstacles that have to be overcome, for both the young adult patient and the parents of the patient (21, 22).

Young adult patients generally consider that they previously knew about their treatment and had a close relationship with their physician and specialist carers. However, at transition they have to get to know a new physician and group of health-care specialists and, therefore, may feel that they are being abandoned by the pediatrician. Pediatric endocrinologists are likely to be more involved with the patient and will explain treatments in simple terms whereas adult endocrinologists are likely to explain treatment in more technical terms that the patient may not understand. Also, a young adult patient attending clinic visits with other patients who are much older, but who might have many more complications associated with the disease, may become fearful of what the future holds.

It is mandatory for physicians and other health-care staff to work with the parents as well as the patients (23), because parents will share the same fears as their children. Parents are likely to have been heavily involved in treatment decisions early in the course of a chronic disease and may have been the first point of contact with the physician in regard to treatment decisions. However, parents now have to be able to let go and allow their child to become independent, although they will still feel protective.

In some countries, such as Canada and the UK, the process of transition involves questionnaires at different stages (19, 21, 23). The aim is to find out what the patient knows about their disease and treatment options, and perspectives of the patient on future progress of the disease. The adult endocrinologist has to discuss various factors, such as autonomy, independence, and sexuality. Autonomy means that the patient has to think about how they can manage their condition, what the day-to-day practicalities are, and which other people need to be involved. The patient may wish to discuss aspects of sexuality and fertility, and adults other than the parents may be needed to help with this. Although perhaps less relevant to GH deficiency, in many chronic diseases risk of death is also a question that frequently requires discussion.

A therapeutic education program has been set up in endocrine units in Paris, France, to assist patients through transition (24). The program has encompassed both individual interviews and group sessions involving both patients and health-care specialists. Patients are included irrespective of their disease, although all are going through transition, with patients aged 16–20 years. Each group session involves six to eight patients for a full day and the patients have to describe their past experience of the disease and its management, as well as how they imagine the future.

The program goals were to identify new hospital structures for patients in transition and the means by which patients can invest in their own health. Following on from this, health-care pathways with specific milestones are being set up for patients in transition. The hospital pathway involves welcoming the patient, organizing administrative structures, and providing therapeutic education. The next step is to provide links from the pediatric department to the adult department, which encompass all aspects of current life of the patient. This provides a space for the patient to discuss what will happen in future, for example in school and in college, and builds up a relationship between the patient and their health-care providers. The program enables use of all aspects of information and communication, using social media and computer applications, and can integrate the patient’s understanding and control of the disease during transition.

## Transition in Practice—A Case Study

There are specific definitions for transition, for example it is defined by the Society for Adolescent Medicine as a purposeful, planned process that addresses the medical, psychosocial, educational, and vocational needs of adolescents and young adults with chronic physical and medical conditions as they move from child-centered to adult-oriented health-care systems (20). However, transition can differ greatly between patients depending on their diagnosis, treatment journey, and capacity; thus, a patient with significant treatment-related late effects may transition at a later time point compared to that of a young person who has minimal long-term effects from their treatment. GH deficiency can arise from multiple etiologies and for patients who develop deficiency following treatment for cancer during childhood, transition is further complicated due to changes in health-care professionals, including endocrine specialists. Thus, there may be more than one transition period for these patients.

A 21-year-old female, with a diagnosis at 5 years of age of rhabdomyosarcoma of the left pterygoid region, received surgery, chemotherapy, and radiotherapy. Scatter of the radiotherapy dose to her pituitary gland resulted in development of pan-hypopituitarism shortly after. She received GH treatment, which was given at more than 2 years after completion of the cancer treatment, in accordance with approved indication guidelines, and also thyroxine treatment. Adrenal insufficiency developed at 13 years of age, which also required treatment, while menses were regular at the time of reporting. Multiple specialists had been involved in the care, including clinical oncologists, speech and language therapists, surgeons, and endocrine specialists. She had profound hearing loss, due to platinum-based chemotherapy agents and radiotherapy, learning difficulties that were compounded by the hearing loss, recurrent ear infections requiring intravenous immunoglobulin infusions, and sicca syndrome with difficulties in swallowing.

When due to move to adult services within a large teaching hospital, this patient and her parents were invited to a specialist evening clinic together with similar patients and their parents. A multidisciplinary team was involved in the clinic, and the aim was to discuss the new environment and to identify concerns. Issues discussed included whether future visits should be in a nurse-led or consultant-led clinic and which other specialists would be needed over time.

The patient had stopped GH treatment on reaching final adult height, but then later presented with central adiposity and low energy levels. In the teenage and young adult transition clinic, further testing of GH status was necessary and, when GH was re-started, further training on administration was required and funding for the treatment had to be identified. There was ongoing regular contact and the patient could continue communication with the transition team for several years while at the same time building a relationship with the adult endocrine team.

Within the UK, multiple sources of information on transition are provided, including policy documents from the Royal College of Nursing (25) and the Department of Health (26). Additionally, a Ready, Steady, Go program from Southampton University Hospital has been adopted by many UK trusts as a transition model (9), whereby patients start to learn about and be involved in transition planning from the age of 11 years, with information constantly updated at each consultation. This allows for a gradual process that gives the patient, their family and the current health-care team time to plan collaboratively and discuss their health-care needs when moving forward into adult health-care settings. In turn, this allows identification of keyworkers, strengthens relationships, and gives the patient control of their health-care journey.

Endocrine experts acknowledge, however, that no single model for transition is going to be universally successful and tailored care is required for patients who require continued GH treatment from childhood through into adulthood. A recent report by an expert group of health-care professionals concluded that there remains much to be done to ensure that the needs of adolescent patients with chronic endocrine conditions continue to be met during transition (1).

## Discussion Session

In response to a question to the audience, multiple participants stated that they ran transition clinics, similar to the UK experience, and that the meeting frequency depended on the number of patients involved. It was noted that several meetings with each patient were required to identify the patient’s knowledge and understanding about their specific condition, to provide a full picture of issues that the patient has and to establish a relationship with the adult endocrinology team. This needed a specified person within the health-care system to manage transition and coordinate the treatment strategy. However, it was also noted that there may be problems with financing a specific person and the model may differ by country according to factors such as reimbursement available and skills of the personnel involved. In Germany, it was reported that in 2008 only 13 of 69 pediatric endocrinologists responding to a structured questionnaire transferred GH-deficient patients in a transition clinic setting (8). However, communication and information transfer was noted to be impaired in endocrine centers without a transition clinic.

The process of transition should start in the pediatric setting, when the patient is around 11–12 years old and should involve sessions both with the child alone and together with the parents. It was suggested that one way to indicate the understanding of the patient was to get them to explain their condition to their relatives. It may also be useful to provide mentors for the patients, who are older patients with the same condition and who can provide explanations from a different perspective. While it should be a partnership with patients, the children and young people need to establish self-management. However, the transition team must be able to identify children who are at risk of life-threatening conditions, such as adrenal insufficiency (27); young patients do not necessarily feel ill and may not recognize risks involved in not managing the condition correctly.

With regard to GH treatment, it is always necessary to explain fully to the patient that continuing with GH is important. This is also important for children diagnosed as GH deficient at older age, even when already into the transition period (28). A significant proportion of patients who remain GH deficient at the end of pediatric treatment do not continue to receive GH during or after transition (29). Patients need a full explanation of the continued effects of GH on lipids, body composition, and quality of life. While protocols for re-testing for GH deficiency at the time of transition have been published (5–7, 16), there continues to be discordance and the normal values for stimulation tests have not been fully established. In response to a question about GH dosing, it was suggested that dose should start at an adult level and then be up-titrated to normalize the IGF-I level. However, it should be noted that females taking concomitant oral estrogen may require a higher GH dose (30). An adequate dose through the transition period is required to attain peak bone mass, and it was also suggested that higher doses may be needed when patients get older because the response to GH may decrease.

## Author Contributions

All authors critically revised the current work for important intellectual content and gave final approval of the version of the publication to be published. All authors have agreed to be accountable for all aspects of the work in ensuring that questions related to the accuracy or integrity of any part of it are appropriately investigated and resolved.

## Conflict of Interest Statement

BH reports non-financial support from Merck-Serono, during the conduct of the study; grants, personal fees, and non-financial support from Pfizer, personal fees and non-financial support from Novo Nordisk, personal fees and non-financial support from Ferring, grants, personal fees, and non-financial support from Sandoz, personal fees and non-financial support from Merck-Serono, personal fees and non-financial support from Ipsen, outside the submitted work. PT has no disclosures. TU-K reports personal fees from Merckgroup outside the submitted work. EK is an employee of Merck KGaA.

## Footnotes

^1^Maghnie M, Labarta JI, Koledova E, Rohrer TR. Short stature diagnosis and referral. Submitted to *Frontiers in Endocrinology*.
